# Review of strategies toward the development of alloy two-dimensional (2D) transition metal dichalcogenides

**DOI:** 10.1016/j.isci.2021.103532

**Published:** 2021-11-29

**Authors:** Appu Kumar Singh, Partha Kumbhakar, Aravind Krishnamoorthy, Aiichiro Nakano, Kishor Kumar Sadasivuni, Priya Vashishta, Ajit K. Roy, Vidya Kochat, Chandra Sekhar Tiwary

**Affiliations:** 1 Metallurgical and Materials Engineering, Indian Institute of Technology, Kharagpur, West Bengal 721302, India; 2 Collaboratory for Advanced Computing and Simulations, Department of Physics and Astronomy, University of Southern California, Los Angeles, CA 90089, USA; 3 Center for Advanced Materials, Qatar University, Doha, Qatar; 4 Materials and Manufacturing Directorate, Air Force Research Laboratory, Wright Patterson AFB, OH 45433-7718, USA; 5 Materials Science Center, Indian Institute of Technology, Kharagpur, West Bengal 721302, India

**Keywords:** Materials science, Materials synthesis, Nanomaterials

## Abstract

Atomically thin two-dimensional (2D) transition metal dichalcogenides (TMDCs) have attracted significant attention owing to their prosperity in material research. The inimitable features of TMDCs triggered the emerging applications in diverse areas. In this review, we focus on the tailored and engineering of the crystal lattice of TMDCs that finally enhance the efficiency of the material properties. We highlight several preparation techniques and recent advancements in compositional engineering of TMDCs structure. We summarize different approaches for TMDCs such as doping and alloying with different materials, alloying with other 2D metals, and scrutinize the technological potential of these methods. Beyond that, we also highlight the recent significant advancement in preparing 2D quasicrystals and alloying the 2D TMDCs with MAX phases. Finally, we highlight the future perspectives for crystal engineering in TMDC materials for structure stability, machine learning concept marge with materials, and their emerging applications.

## Graphical abstract

**Figure:**
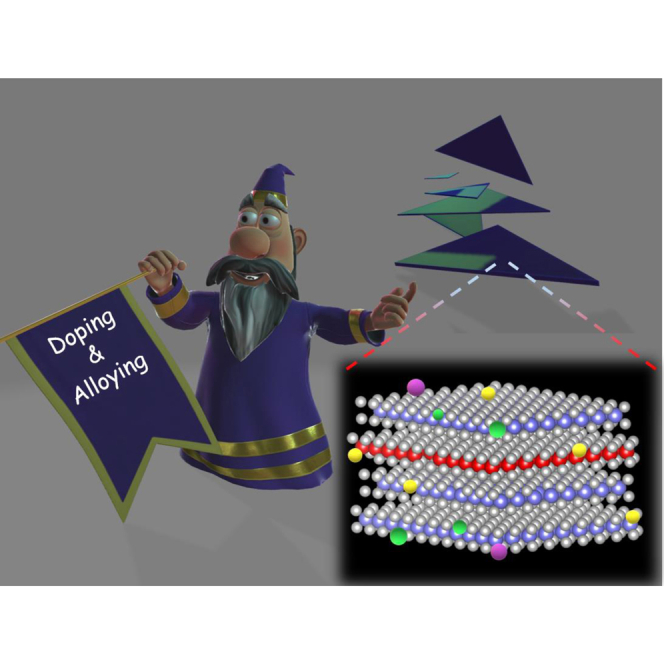

## Introduction

After discovering graphene, an impressive improvement in fundamental science and technology implementation has been explored using two-dimensional (2D) materials. Over the past few years, a broad range of atomically thin 2D materials, for example, graphene-based 2D materials, transition metal dichalcogenides (TMDCs), transition metal carbides and nitrides (*MXenes*), layered oxides, 2D metal-organic frameworks, and their layered derivative structures, has been prepared owing to their novel structural properties, extensive applications in electronics, optoelectronics, energy generation/conversion, biomedical technology, and catalysis (Anasori et al., 2017; George et al., 2019; Kochat et al., 2017, 2018; Kumbhakar et al., 2021). Most significantly, 2D layered materials show several physical properties ranging from insulator to narrow-gap semiconductor to semimetal or metal. Multiple structural phases have been observed at the molecular level owing to the variations of crystal configurations. Different synthesis method has been employed to prepare these 2D materials as summarized in several reviews (Mannix et al., 2017). Synthesis strategies for 2D materials can be classified into two broad groups: top-down and bottom-up methods. Among them, mechanical exfoliation, liquid exfoliation, ion-intercalation, chemical vapor deposition (CVD), wet-chemical synthesis, and so on are suitable for producing 2D materials on a large scale. However, for the synthesis of 2D materials, the synthesis parameters are crucial such as in mechanical exfoliation, the peeling methods, mechanical properties of the materials are crucial. In the liquid-phase exfoliation method, the most critical parameters are the choice of solvent because of the surface energy, which can control the exfoliation and aggregation of sheets. In the CVD process, controlling the temperature, precursor ratio, substrate, and gas flow rate also affects the morphology of the synthesized material. Therefore, these traditional synthesis methods have some shortcomings.

Despite the significant potential of 2D materials, controlling the structural, physical, chemical properties, optimization of materials compositions, tuning of defects, etc., are the most daunting challenges for the materials field. Different approaches such as alloying, doping, mixing of multiple atoms, functionalization, etc., have been employed in this context. Owing to the weak van der Waals (vdW) interaction between layers, layered TMDCs with different structural modifications have attracted extensive attention. Recently, creating defects by removing specific atoms on the TMDC structures show many fascinating optoelectronic applications (Yin et al., 2021). The insertion of foreign atoms (dopant) has altered the properties of layered TMDCs. Apart from doping, the engineering of compositions has been demonstrated to be an active method for resolving the challenges.

**Figure 1:**
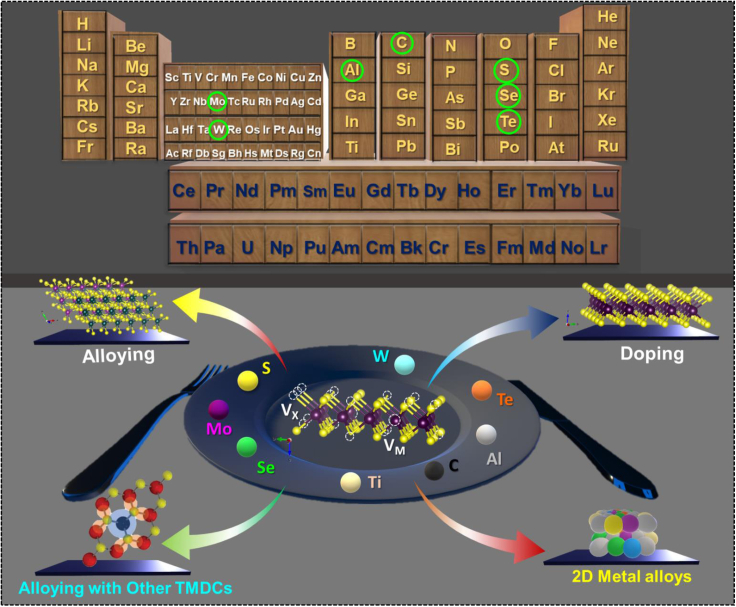
Schematic presentation of different types of 2D TMDCs materials and their preparation methods

To date, several reviews have discussed the modern synthesis techniques of 2D materials and their potential applications. However, compositional engineering in 2D TMDCs, such as doping with different metals, alloying with other TMDCs and *MXenes*, has not been explored so much. This review mainly focuses on the recent progress related to developing novel 2D TMDCs materials and their potential applications. Here, we have discussed the doping of several metal ions in the TMDCs crystal. The used doping techniques for the characterization of the materials are systemically summarized in Figure 1. Then, we discuss alloying of 2D TMDCs with other materials; it can be classified into four subsections: metal replacement, dichalcogenide replacement, metal and dichalcogenide replacement, and multicomponent replacements. The MAX phases are polycrystalline nanolaminates of ternary nitrides or carbides, and their alloying with 2D TMDCs is discussed extensively. After that, the working mechanism of doping/alloying materials has been discussed in detail. After such a detailed analysis of the progress of 2D TMDCs, some challenges and perspectives on the further development of high-quality 2D TMDCs explore. The discussions provide a theoretical basis and technical support for implementing other functional strategies and could be extended to other 2D TMDCs-based optoelectronic devices.

## Results and discussion

### Characteristics of doped 2D transition metal dichalcogenide

2D TMDCs can be expressed as MX_2_, M is a transition metal element such as Mo, Ta, W, and Nb, and X is S, Se, and Te. Doping in 2D TMDC is achieved by either replacing the M site, X site, or interstitial site with a foreign atom. Approach reportedly helps to tune the material to achieve desired characteristics such as better thermal, electrical conductivity, photosensitivity, controlling phonon dispersion, etc. In Table 1, we have summarized the doping of various atoms in 2D TMDCs.

**Table 1:** Substitutional doping in transition metal dichalcogenide

Host	Doping Site	Dopants	Dopant group	Dopant type	Doping conc.	Doping synthesis Method	Ref.
MoS_2_	Mo	Re	7	n-type	2.1 atomic %	Molten salt CVD	(Li et al., 2021; Zhang et al., 2020a)
WS_2_	W	Fe	8	n-type	0.7–2.8 atomic %	Liquid precursor-assisted technique	(Zhang et al., 2020a, 2020b)
NbS_2_	Nb	Cu	11	p-type	0–1.2 atomic %	Powder metallurgy	(Liu et al., 2020)
WS_2_, MoS_2_, WSe_2_, MoSe_2_	Mo, W	Rb	1	n-type	∼ 10^12^ cm^−2^	In-situ surface doping	(Kang et al., 2017)
WSe_2_, MoS_2_, WS_2_	Mo, W	V	5	p-type	2.7–5 atomic %	CVD	(Li et al., 2021; Williamson et al., 2019)
MoSe_2_, MoS_2_, MoSe_2_	Mo, W	W	6	p-type	2.083 atomic %	–	(Williamson et al., 2019)
NbS_2_, NbSe_2_	Nb	Li,Na	1	n-type	0.125 atomic %	–	(Fan et al., 2017)
MoS_2_, MoSe_2_, WS_2_, WSe_2_	Mo, W	Li,Na	1	n-type	0.125 atomic %	intercalation using powder metallurgy route	(Fan et al., 2017; Onofrio et al., 2017)
MoTe_2_, WSe_2_, MoSe_2_, PtSe_2_, PdSe_2_	Mo, W, Pt, Pd	O	16	p-type	∼ 10^13^-10^14^ cm^−2^	Surface doping	(Liang et al., 2020)
MoS_2_, ReS_2_	Mo, Re	Al_x_O_y_	–	n-type	1.6–1.8 atomic %	Encapsulation by atomic layer deposition.	(Leonhardt et al., 2019)
WS_2_	W	Al_x_O_y_	–	–	1.6–1.8 atomic %	Encapsulation by atomic layer deposition.	(Leonhardt et al., 2019)

**Figure 2:**
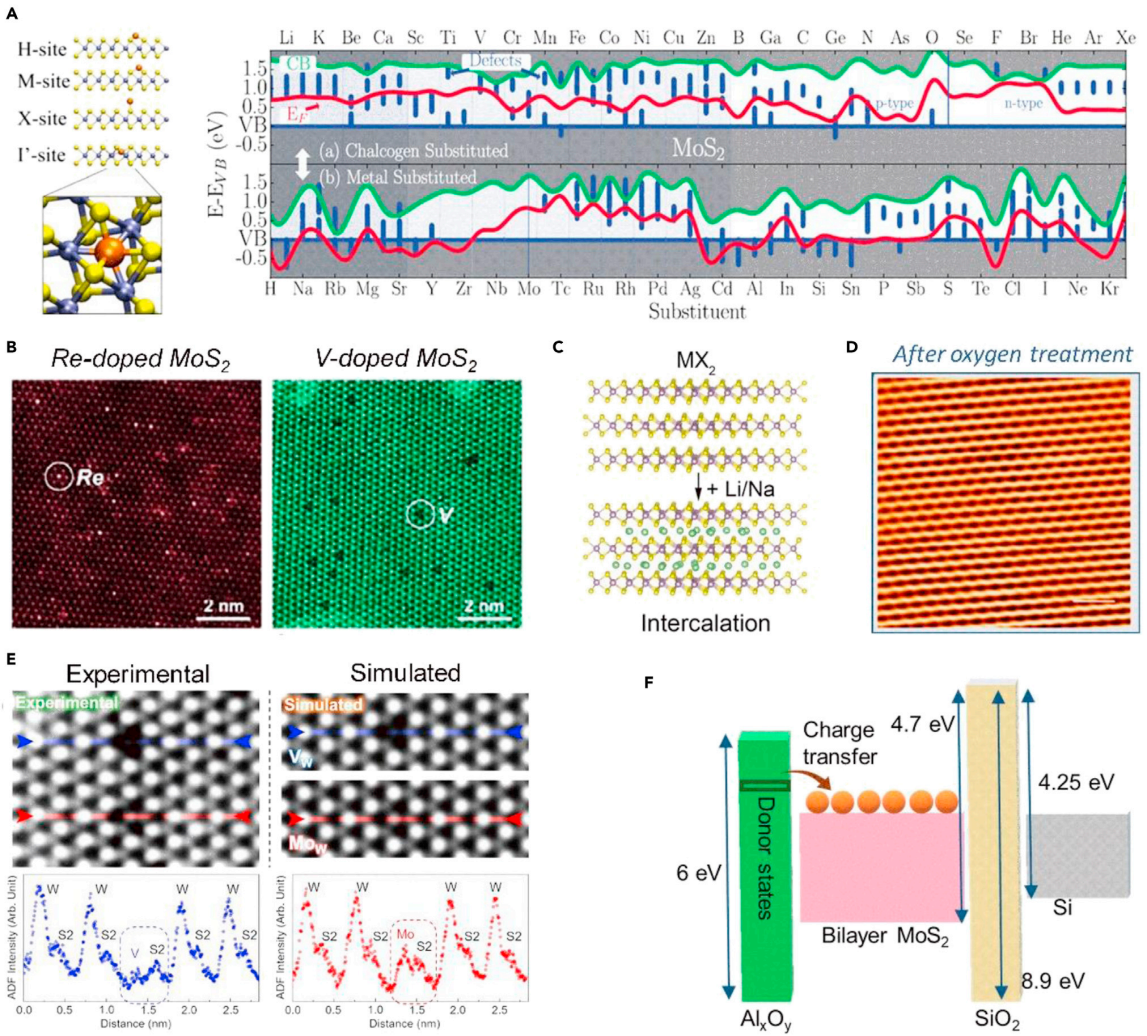
Prospects of periodic element substitution in 2D TMDC (A) The energy band diagram of monolayer MoS_2_ as a function of dopants (Onofrio et al., 2017). (B) STEM images at 25% doping ratio for Re-doped MoS_2_ (left) V-doped MoS_2_ (right) monolayers (Li et al., 2021). (C) Schematic of the structure of MX_2_ with intercalation sites. Transition metal atoms are in purple, chalcogen atoms in yellow, and intercalated Li/Na atoms are in green spheres, respectively (Fan et al., 2017). (D) STM image after oxygen treatment and signifying the oxygen doping has no impact on the host crystal structure (Scale bar 1 nm and V_S_ = 700 mV) (Liang et al., 2020). (E) (Right) Fe_W_ and 3Fe_W_ + V_S_ dopant configurations' experiment (dotted lines) and calculated(solid) STEM images and line scans; (Left) Re-doped MoS_2_ (Zhang et al., 2020a). (F) Schematic diagram representing charge transfer behavior with Al_x_O_y_ deposition on MoS_2_ (Leonhardt et al., 2019).

Experimental justification and theoretical studies suggested that substitutional doping of Group I to II in MX_2_ will result in an n-type semiconductor with ∼7%–18% lattice expansion, mostly in the c-direction of the lattice which may indicate the phase transformations. Bulk MX_2_ was treated with n-butyl lithium (n-Bu Li) to obtain doped MX_2_ with alkali metals. Purified Li_x_MX_2_ was exfoliated in deionized water by ultrasonication. Lithium intercalation yields 90% *1T′* MX_2_ after exfoliation and gets transformed to *1T* and *2H* structure under electron beam microscopy imaging owing to its instability. Bond formation energy predicts the movement of atoms in lattice and final stable structure of new alloy. Bond formation energy of Li with substitutional MX_2_ on M site or X site was greater than the bond formation energy in interstitial site. Doping of lithium and sodium in MX_2_ resulted in an n-type semiconductor with lattice expansion, for which theoretical studies suggests that intercalation of alkali metals in MX_2_ octahedral site is more favorable than tetrahedral site because of higher coordination number and approximately three times larger space. Intercalation in WX_2_ lattice also showed increment in interlayer spacing, caused by the change in phase from *2H* to *1T* phase and Fermi level shift toward up, *i.e.*, valence band maximum to the conduction band (Fan et al., 2017; Onofrio et al., 2017). Similar phenomena were observed for MX_2_ lattice where phase transformation of MX_2_ from *2H* to *1T* is also supported by experimental justification by synthesis of Li intercalation using powder metallurgy route (Figure 2C) (Padmajan Sasikala et al., 2020). For NbX_2_ and TaX_2_, no such phase transfer occurs, but their Fermi level showed moving into a gap that is not suitable for electron transport (Fan et al., 2017). In-situ surface doping of rubidium (Rb) atoms in *2H* TMDCs (WS_2_, MoS_2_, WSe_2_, and MoSe_2_) resulted in n-type semiconductor of 0.8–2 eV bandgap for concentration of ∼ 10^12^ cm^−2^. Valence and conduction band get progressively closer owing to ionized Rb atoms on the surface of *2H*-TMDCs. Rb-doped TMDCs reported causing structural changes because of potential difference between the layers that break inversion symmetry and leads to spin splitting of valence bands (Kang et al., 2017).

Furthermore, for Group III elements, the theoretical investigation suggested doping of Ni, Sc in MX_2_ lattice may result in the distorted lattice as much as ∼12% lattice size reduction with MX_2_ lattice and with tellurides exhibiting even more significant distortion than other dichalcogenides due to *1T′* phase transformation. Sc-doped MX_2_ lattice was semiconducting material with a suggested bandgap of 0.14 eV, whereas Ni was the least stable dopant. It is being estimated that the Sc's atomic radius plays a critical role in its stability than its oxidation state. Recently, Williamson et al. and his group reported that W or Mo doping in MX_2_ lattice has minimal impact on the density of state as the difference in atomic radii and oxidation state was near equal, resulting in p-type doping (Williamson et al., 2019).

Substitutional doping of transition elements in 2D TMDCs from group IV to group XII results in better dopants. There is a negligible distortion because of similar atomic radii of element and nearby oxidation state. The substitutional doping of Vanadium (V) in WS_2_ and WSe_2_ results in p-type with negligible lattice distortion, approximately the same lattice parameters, and low contact resistance. Doping of V in MoS_2_ was reported as poor p-type conduction behavior than WS_2_ and WSe_2_ as the doped sample was determined to have a degenerate state (Figure 2B) (Li et al., 2021; Williamson et al., 2019). Doping of Fe in WS_2_ lattice resulted in n-type with defect coupling and lattice distortion similar to Mo-doped WS_2_ lattice (Padmajan Sasikala et al., 2020). Doping of Fe in MoTe_2_ lattice-induced spins on nearby Te and Mo atoms is parallel to dopants. Similar phenomena were observed for Cr, Mn, Co, and V doped in MoTe_2_ exhibiting magnetic properties, and Ni doping exhibited non-magnetic properties (Kanoun, 2018; Tiwari et al., 2021). The atomic doping changes the band structure of MoS_2_ layers, and new defect states are introduced near conduction band minima (Figure 2A). The approach was attempted by the CVD method – VLS growth of layers using molten salts, which was also found to be applicable in liquid precursor-assisted technique of *in situ* substitutional doping (Li et al., 2021; Zhang et al., 2020a). Cu intercalation into 2D TMDC was 1.2 atomic % in NbS_2_. Substitutional doping of Cu in a tetrahedral site is coordinated by S atoms, where doping leads to an upshift Fermi level in-band structure with a 0–0.65 eV bandgap. Similar Cu doping in Group IV and V TMDC *2H* led to no Cu species are intercalated into group VI TMDC, such as MoS_2_ or WS_2_ (Liu et al., 2020). Most significantly, substitutional doping of Group XIII to XVI mostly resulted in p-type semiconductors, whereas doping of Group XVII elements resulted in n-type doping, as shown in Figure 2A (Onofrio et al., 2017). Surface doping of oxygen resulted in p-type semiconductor in TMDCs (MoTe_2_, WSe_2_, MoSe_2_, PtSe_2_, and PdSe_2_) for concentration of ∼ 10^13^–10^14^ cm^−2^ (Figure 2D). Isoelectronic substitution of chalcogen atoms, Se to O atoms, induces trapping potential due to electronegativity difference resulting in modulation of free carrier density and conduction type (Liang et al., 2020). Doping of Re, in MoS_2_, resulted in n-type with lattice scattering and distorted *1T* phase structure. Monolayer CVD growth was performed at 750°C. Salt precursors mixing were in designated ratios in a tube furnace. As the doping concentration increased, more distortion has been reported contributing to the ReS_2_ structure that increased degenerate electron transport and improved conductivity at 2.1 atomic % Re-doping. Leonhardt et al. highlighted the importance of band alignment by demonstrating low-temperature atomic layer deposition of Al_x_O_y_ in MoS_2_ (Figure 2F) (Leonhardt et al., 2019). Atomic layer deposition of Al_x_O_y_ on using tetra methyl aluminum and H_2_O precursors results in high doping on synthetic MoS_2_-exfoliated flakes. This approach resulted in electrons donation as the conduction bands' edge of MoS_2_ lies at similar or lower energy than Al_x_O_y_ donor states; it is energetically favorable for electrons from donor states to move into acceptor states of MoS_2_, ReS_2_, and converting them into n-type semiconducting material. This result was not applicable for WS_2_ as the WS_2_ conduction band lies at higher energy, and electrons cannot be donated.

### Characteristics of 2D transition metal dichalcogenide alloys

**Figure 3:**
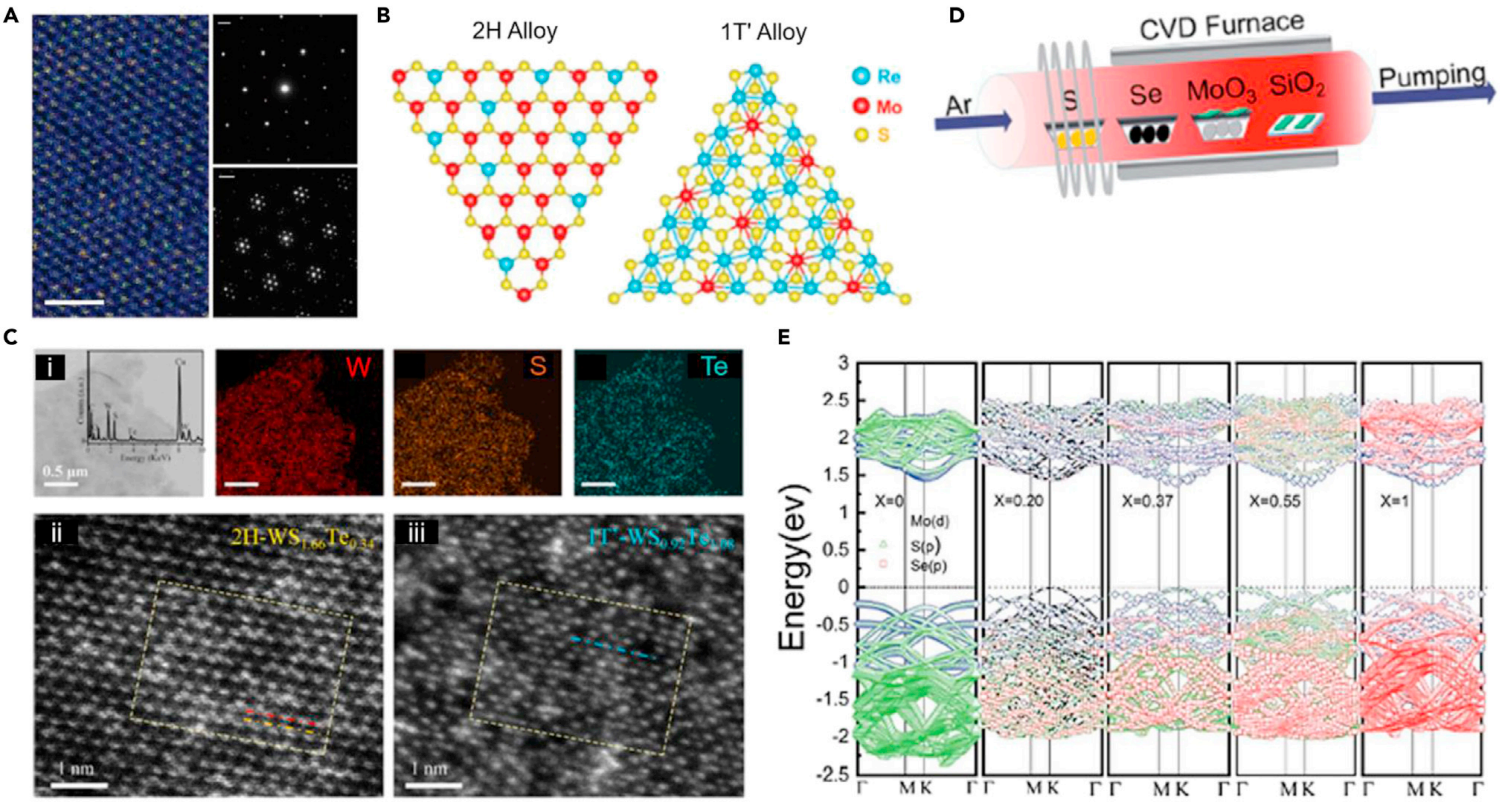
Application of Alloying in 2D TMDC (A) The atomic-resolution HAADF of Mo_(1–x)_Nb_x_Se_2_ (left, scale bar: 1nm), and diffraction patterns from 2H (top, scale bar: 2 nm^−1^) and 1T Mo_(1–x)_Nb_x_Se_2_ flakes (bottom, scale bar: 2 nm^−1^), respectively (right) (Wang et al., 2020a). (B) Atomic structure of 2H and 1T′ Mo_x_Re_(1–x)_S_2_ alloys (Deng et al., 2020). (C) TEM characterizations of 2H and 1T′ phase alloys. The insert shows the TEM-EDX survey spectrum—element mappings of W, S, and Te from WS_1.66_Te_0.34_. (ii-iii) HAADF-STEM images of monolayer 2H WS_1.66_Te_0.34_ and 1T′ phase WS_0.92_Te_1.08_ alloys (Wang et al., 2020d). (D) Schematic of synthesis method of TMDCs alloy (E) MoS_2(1-x)_Se_2x_ Band energies at x ¼ 0, 0.20, 0.37, 0.55, and 1 (Yao et al., 2020).

Alloying is a powerful technique to tailor the structures in order to tune the desired property. Alloying of 2D TMDCs has gained vast popularity over the past few years. Even so, they have several drawbacks, including the synthesis of these structures at a large scale. The alloys can be classified into four subsections: metal replacement, dichalcogenide replacement, metal and dichalcogenide replacement, and multicomponent replacements. We will be discussing briefly on each of these sections. The structure control during material synthesis, which involves thermodynamic stability, is crucial during alloying 2D chalcogenides. 2D materials also face the challenge of being unstable and highly reactive compared to their bulk counterpart. 2D TMDCs reportedly have bipolar behavior, and significant band engineering can be approached with substituting alloying components. Various reported alloying attempts had been summarized in Table 2. Most of the alloying is carried about by CVD method, process starts with mixing powders in stoichiometric ratio; growth of bulk crystals inside a quartz tube is placed in furnace and followed by standard mechanical exfoliation to obtain monolayer flakes. Alloying in the metal component reported increasing p-type behavior of 2D TMDCs with few distortions in the lattice. Alloying W in Mo site, Mo_(1-x)_W_x_X_2_, where X is S, Se reported to have 0.268 eV and 0.167eV owing to bond-stretching effect as the contribution is attributed to the Coulomb electrostatic force as the anion undergoes an offset when the cation is doped. The study may imply the growing band shift with an increase in the dopant composition (Dong et al., 2019). While alloying Mo in W site, Mo_x_W_1-x_Se_2_ showed p-type characteristics with a bandgap of 1.44–1.53 eV. As the Mo concentration is increased, valence band dispersion and spin-splitting size are monotonically reduced. Hence, it highlighted the importance of controlling stoichiometric ratio in-band tuning (Xie et al., 2020). Another approach (Wang et al., 2020a) explained the phase-dependent characteristics of Nb alloyed in Mo site, Mo_(1-x)_Nb_x_Se_2_, where *2H* phase exhibiting metallic behavior and *1T* phase with distortion cause semiconductor behavior with a bandgap of ∼0.4 eV. Figure 3A shows the phase mixture state of Mo_(1-x)_Nb_x_Se_2_.

**Table 2:** Alloying in Transition metal di-chalcogenides

Alloy	*x* (Conc.)	Bandgap (eV)	Semiconductor Type	Synthesis Method	Ref.
MoS_2(1-x)_ Se_2x_	0-1 atomic %	1.5–1.8	p-type	CVD	(Yao et al., 2020; Dong et al., 2019)
Mo_(1-x)_W_x_S_2_	0-1 atomic %	1.8–2.01	–	–	(Dong et al., 2019)
Mo_(1-x)_W_x_Se_2_	0-1 atomic %	1.5–1.7	–	–	(Dong et al., 2019)
WS_2(1-x)_Se_2x_	0-1 atomic %	1.7–2.01	–	–	(Dong et al., 2019)
WS_2x_Se_2(1-x)_	0.48 atomic %	3.5–3.9	p-type	CVD	(Ko et al., 2018)
WS_2(1-x)_Te_2x_ (*2H*)	<0.5 atomic %	1.97–1.67	p-type	CVD	(Wang et al., 2020d)
WS_2(1-x)_Te_2x_ (*1T*′)	≥0.5 atomic %	0	n-type	CVD	(Wang et al., 2020d)
Mo_x_Re_(1-x)_S_2_ (*2H*)	<0.3 atomic %	–	n-type	CVD	(Deng et al., 2020)
Mo_x_Re_(1-x)_S_2_ (*1T*′)	≥0.3 atomic %	–	p-type	CVD	(Deng et al., 2020)
Mo_x_W_(1-x)_Se_2_	0-1 atomic %	Direct, 1.44–1.53	p-type	MBE	(Xie et al., 2020)
Mo_(1-x)_Nb_x_Se_2_ (*1T*)	0.5 atomic %	0.42–0.58	p-type	CVT	(Wang et al., 2020a)
Mo Se_2(1-x)_Te_2x_	0.1–0.5 atomic %	0.945–1.5	–	CVD	(Apte et al., 2018; Kanoun and Goumri-Said, 2020)
Mo_x_W_(1-x)_S_2y_Se_2(1-y)_	0-1 atomic %	1.61–1.85	p-type	CVD	(Susarla et al., 2017)
MoS_2_/Mo_(1-x)_W_x_Se_2_	0-1 atomic %	0.34–0.67	p-type	–	(Zi et al., 2019)
Mo_(1-x)_W_x_S_2_/MoSe_2_	>0.57 atomic %	Indirect, 0.67–0.68	n-type	–	(Zi et al., 2019)
	<0.57 atomic %	Direct, 0.72–0.83	p-type	–	(Zi et al., 2019)

Alloying in chalcogen component reported having n-type, p-type, or bipolar behavior characteristics. When alloying in Te in chalcogen site, WS_2(1-x)_Te_2x_ reportedly showed bipolar behavior with structural change from *2H* phase to *1T*′, when Te alloyed with chalcogen site. Less than 0.5 atomic % of doping, material reported as p-type semiconductor while more significant than 0.5 atomic % resulted in n-type (Figure 3C). The root cause of the phase transition behavior is mainly attributed to the nature of Te for stronger metallicity and higher melting point (Wang et al., 2020d). Similar phenomena can be observed as bipolar behavior when alloyed in the metal site of 2D TMDC for Mo_x_Re_(1-x)_S_2_ (Deng et al., 2020). Mo Se_2(1-x)_Te_2x_ reportedly has a stable *2H* structure, but alloying of telluride, Te, increased the electron effective mass in-band structure mainly because Te showed metallicity characteristics (Figure 3C) (Apte et al., 2018; Kanoun and Goumri-Said, 2020). Ko et al. demonstrated that alloying sulfur, S, in chalcogen site in WSe_2_ results in enhancing NO_2_ gas sensing properties by 2.5 times from WSe_2_ (Ko et al., 2018). Alloying Se in chalcogen site, Mo S_2(1-x)_ Se_2x_ resulted in p-type semiconductor for 0 to 1 atomic % with a bandgap of 1.82 to 1.53 eV using low-pressure CVD method (Figure 3D). Bandgap values decrease with Se doping in MoS_2_ lattice as the Se occupies the highest occupied molecular orbital and lowest unoccupied orbital (Figure 3E) (Yao et al., 2020). It is also reported to have less distortion energy of 0.06 eV, resulting in the valence band orbital and conduction band orbital having an increase or decrease monotonically with the dopant composition (Dong et al., 2019).

Alloying in both metal and chalcogenide sites may show p-type semiconducting behavior. Susarla et al. demonstrated material optical properties with alloying, Mo_x_W_(1-x)_S_2y_Se_2(1-y)_, with CVD synthesis (Avdizhiyan et al., 2021; Susarla et al., 2017). CVD Synthesis of the quaternary alloy at 750°C provided a better distribution of composition throughout the sample with a bandgap range of 1.61–1.85 eV. Interestingly, if the quaternary alloy has a miscibility gap, then annealing may result in the heterostructure, as demonstrated for Mo_(1-x)_W_x_S_2(1-y)_Se_2y_ that resulted in MoS_2(1-x)_Se_2x_/WS_2(1-y)_Se_2y_ (Susarla et al., 2018).

The theoretical investigation suggested that MoS_2_/Mo_(1-x)_W_x_Se_2_ heterostructure was reported as p-type while Mo_(1-x)_W_x_S_2_/MoSe_2_ heterostructure reported being bipolar behavior transitioning from indirect to direct band structure. W_(1-x)_Mo_x_S_2_/MoSe_2_ heterostructure resulting in n-type when doping concentration is less than 0.57 atomic %, with increasing doping concentration resulted in a p-type semiconductor. Doping mechanism is similar to the other alloy formation. The change in stoichiometric ratio during the compound formation has a significant impact on the electron transfer and availability of states during bonding. As the doping concentration increased, it induces gap in the state which indicates p-type behavior. Similarly, as the doping concentration decreased, the density of state increased at fermi level indicating stronger hybridization and n-type behavior. The underlying cause of bipolar behavior is attributed to the doping lowering of Mo “*d*” orbital energy in the WS_2_ layer (Zi et al., 2019). This study can be scaled for the band engineering tuning of TMDC heterostructures as a function of composition and compounds interlayer coupling.

**Figure 4:**
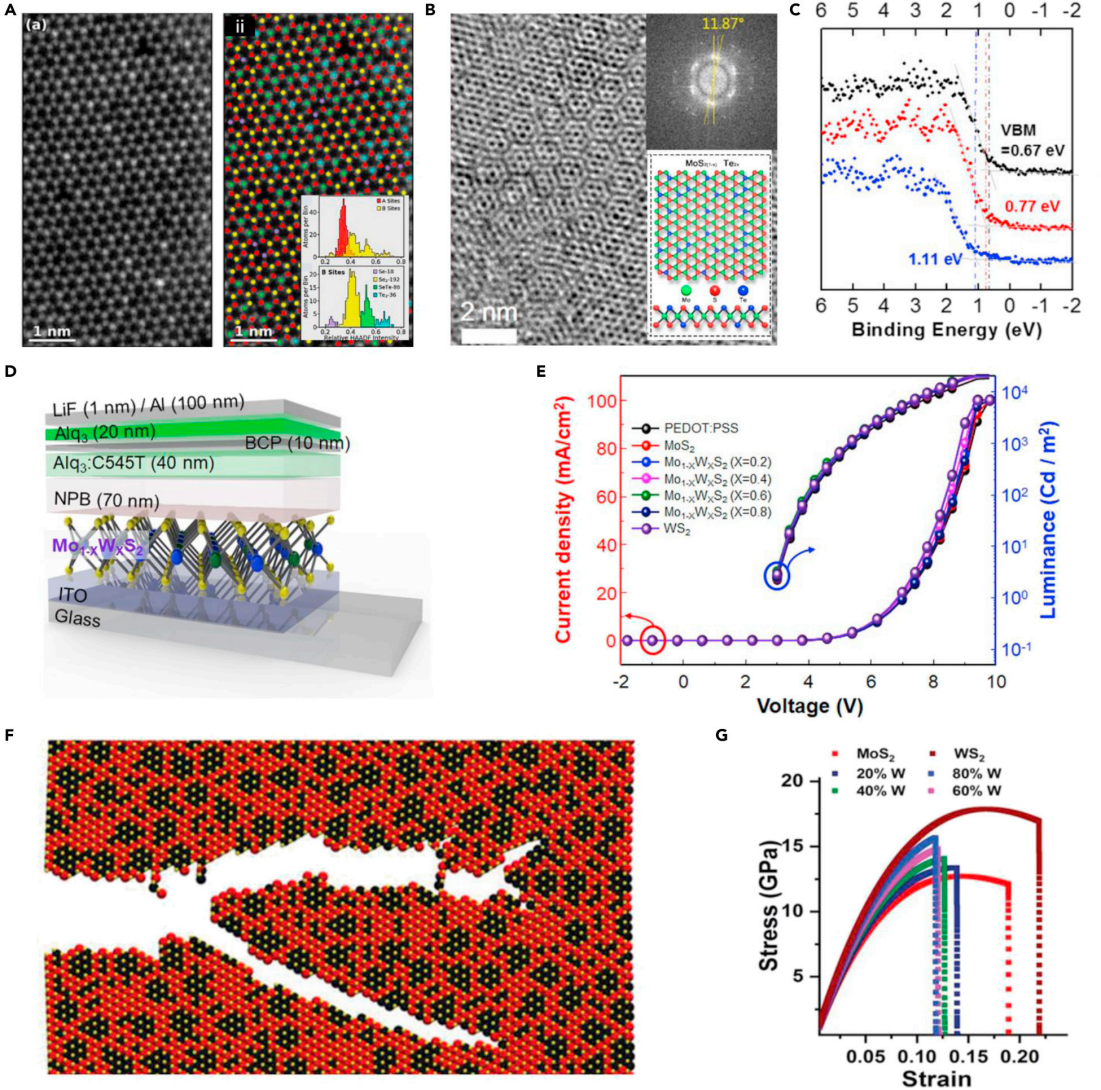
Effect of alloying in 2D TMDC (A) HAADF image of monolayer alloy samples and confirm the successful alloying. Inset shows the presence of atoms in the alloy (Apte et al., 2018). (B) HRSTEM image of MoS_0.8_1Te_1.19_. Inset shows the atomic structure of the alloy. (C) The shifting of VB of MoS_2x_Te_2(1−x)_ as a function of S (Kim et al., 2020) (D) Device structure of OLED. (E) Current density and luminance–voltage curve of the fabricated OLEDs (Kwon et al., 2021). (F) Theoretical result during crack propagation with 40% of W alloy. (G) Biaxial stress vs. strain curves (Susarla et al., 2019).

Molybdenum systems have been widely explored for their stability to form alloys. In a recent study, Apte et al. alloyed 2D monolayer of molybdenum by CBD method with a selenide–telluride combination. HAADF image of MoSe_2(1-x)_Te_2x_ system is shown in Figure 4A, where the semiconducting 2H phase was observed. The 2H phase is isotropic, and the images confirmed the atomistic nature of the alloy formed. The increased tellurium concentration of Se: Te in 1:5 ratios (Alloy B) resulted in a redshift of emission spectra to 1.42 eV. The induced structural change in the 2D monolayer provides an opportunity for optical properties like absorption and emission spectral ranges to be efficiently engineered in 2D TMD alloys (Apte et al., 2018). In a similar study, large area of MoTe_x_Se_2-x_ alloy was formed by the molten salt-assisted CVD method. The photoluminescence spectra analysis showed a 1.55 to 1.38 eV shift in bandgap with increasing tellurium concentrations; thus, finding applications in near-infrared device applications (Zhao et al., 2019b). Studies have shown that Te alloying in excess leads to phase transition from 2*H* to 1*T′* in WSe_2(1-x)_Te_2x_ system, which is not favorable (Yu et al., 2017). There are instances where tuning in between sulfide and selenide bandgap range for molybdenum compositions during the alloying of the MoSSe system (Mann et al., 2014). The tunable bandgap of such systems ranges from 1.5–2.0 eV (Fu et al., 2015; Lin et al., 2017; Wu et al., 2017).

In the subsequent study, the Se-Te combination was replaced with sulfide–tellurium. The thickness of the layers was ∼1.52 nm (three atomic layers), which lies between the monolayer thickness of MoS_2_ and MoTe_2_. Figure 4B shows the high-resolution STEM images of the MoS_0.81_Te_1.19_. Inset shows the rotation between two basal planes by 11.87°, resulting in overlapping these honeycomb structures. Figure 4C shows the dependence of valence band structure on the composition of the material. The composition changes of MoS_2x_Te_2(1−x)_ from x ≥ 0.93 to x ≤ 0.8 resulted in a shift in band structure from n-type to p-type (Kim et al., 2020). We also observe the usage of such alloys as a light-emitting diode. Significant area synthesis of Mo_1-x_W_x_S_2_ as a hole transport layer (HTL) was realized for organic light-emitting diodes (OLED). Figure 4D shows the schematic representation of the fabricated OLED device, which was comparable with the already existing PEDOT: PSS units (Kwon et al., 2021). Figure 4F demonstrates the current density and luminance–voltage curve for the OLED device. The highest luminance of 22,000 cd/m^2^ was achieved using Mo_0.2_W_0.8_S_2_ as HTL, comparable with PEDOT: PSS. As the alloys are favorable in flexible electronic devices, they were also studied for their mechanical performance. The material behavior on extreme stress, strain, and fracture conditions plays a crucial role. Susarla et al. studied the strain-induced structural deformation in 2D Mo_x_W_(1-x)_S_2_ alloy. Figure 4E shows the snapshots taken during MD simulation depicting crack propagation in the TMDCs alloy. The increase in weight content to 40% reduces the failure strain. The study also shows that the weakness is at the interface of the two transition metals and not the MoS_2_ matrix. Figure 4F shows that the weight concentration has a major impact on the fracture limit, as seen in these biaxial stresses vs. strain curves (Susarla et al., 2019). Therefore, exploring alloys in between different chalcogens with different *d*-electrons can help in tuning the bandgap of the 2D TMDCs and also understand their structural limitations. Forming alloys of metals and chalcogens at the 2D level is challenging rather than forming them in a 3D structure as they form non-directional atomic bonding. They are also energetically favored in bulk alloys.

### 2D metals alloy

Tuning of 2D crystals is dependent on the synthesis route taken to achieve the maximum attainable potential-desired properties. Various attempts have been made for 2D crystal synthesizing from their precursors using electron beam irradiation, etching, mechanically straining, or thermal decomposition. Synthesizing with electron beam irradiation using STEM highlights the relation between controlling beam intensity and changing the material's morphology. If the electron beam is controlled optimistically, irradiation can help synthesize 2D crystals from their precursor. This method induces the loss of atoms that can facilitate structural transformation, as it provides enough energy to overcome the barrier for migration leading to vacancy creation (Elibol et al., 2018; Zhu et al., 2017). In the case of amorphous material, with *in situ* heating, electron beam irradiation can induce mobility of atoms resulted in inversion domain grain boundaries that can shift to allowing the variation of inversion domain size. These domains are crystalline, restructured from amorphous material, and are limited to the formation of voids near inversion domains. As the void size grows under prolonged exposure to electron irradiation, inversion domains shrink (Bayer et al., 2018; Chen et al., 2019).

**Figure 5:**
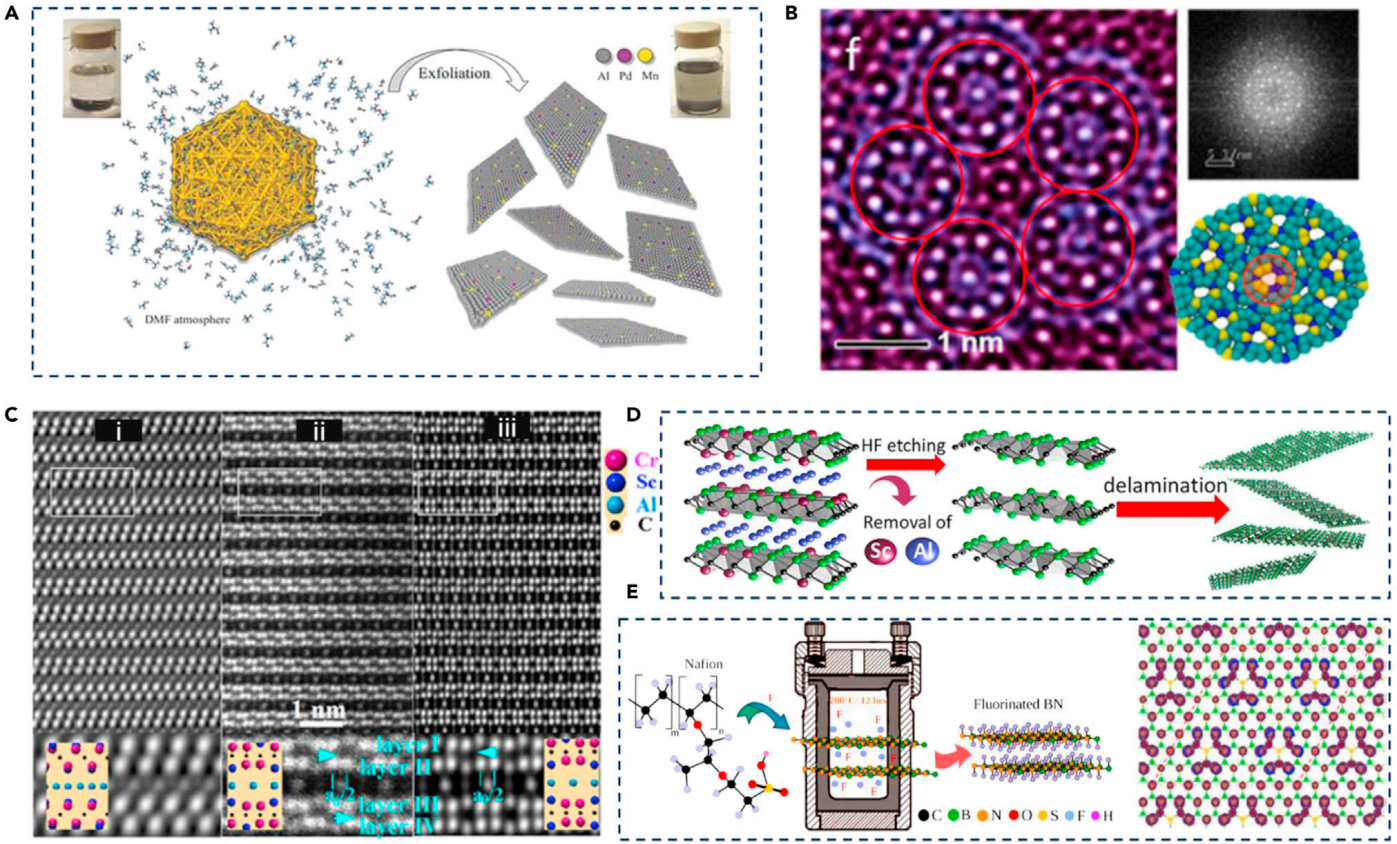
Evolution of 2D metal alloys (A) Graphical representation of 2D Al_71_Pd_20_Mn_9_ decagonal QC synthesis in DMF (Yadav et al., 2020). (B) High-resolution TEM image of exfoliated 2D decagonal QC. Inset shows the ten-fold symmetry with the FFT image of the sample (Yadav et al., 2020). (C) STEM images of (Cr_2/3_Sc_1/3_)_2_AlC-sg15 along (i) [100], (ii) [010], and (iii) [110] plane (Lu et al., 2017). (D) Schematic representation of the synthesis of Nb_1.33_CT_x_ (Halim et al., 2018) (E) Graphical presentation of the synthesis process of fluorinated h-BN (Radhakrishnan et al., 2017).

Similarly, bulk materials having a layered structure can also lead to 2D crystal formation using selective electron beam ionization. This approach may help remaining atoms retain parent crystal structure as bonding between atoms is easy to dissociate (Zhao et al., 2018). However, not every 2D crystal can retain its parent identity (Lin et al., 2017), demonstrated the scotch-tape method to synthesize 2D crystal structure originating from bulk PdSe_2_ layered structure by introducing Se vacancies in PdSe_2_ via electron irradiation. Interestingly, the formation of 2D structure resulted in Pd_2_Se_3_ and did not resemble the PdSe_2_ crystal structure. This deviation from most of the reported literature trends (Zhu et al., 2021) may be due to Pd-Se's strong interlayer interaction. Electron beam irradiation by STEM, Ad-atoms diffusion, and re-arrangement can also lead to 2D crystal formation. Adatoms preferentially occupy sites with more surrounding atom columns and vacancy sites with the movement of atoms for repair governed by controlling beam intensity (Sang et al., 2018; Shen et al., 2018). Another approach for 2D crystal formation has been approached by thermal decomposition of salts. Providing *in situ* heating with STEM helped form short-range order from amorphous salt, as the thermolysis temperature helps develop larger grain size with a layered structure (Sang et al., 2019; Zhang et al., 2020). Yadav et al. highlighted that the 2D metallic alloys' exfoliation from the bulk materials could be difficult due to isotropic forces using a bottom-up approach (Yadav et al., 2018). The study suggested a novel approach synthesizing 2D Quasicrystal (QC) using N-dimethyl-formamide (DMF) solvent, and ultrasonification-assisted exfoliation method (Figure 5A). The novelty of the approach was demonstrated by exfoliation of Al-Pd-Mn poly-grained icosahedral QC and explored its application for electro-catalytic performance where the overpotential was lowered to ∼100mV. The increment in hydrogen evolution reaction performance is mainly because of the morphology change resulting in the presence of active catalytic atoms. In the latter study, similar phenomena were demonstrated for Al-Co-Cu. Firstly, 2D QC was exfoliated and exploring its further application; at the second step, 2D QC was synthesized with WS_2_ to make heterostructure (Figure 5B). The 2D QC/WS_2_ showed that overpotential was lowered to ∼60mV (Yadav et al., 2020).

### Alloying with MAX

The MAX phases are defined as M_n+1_ AX_n_, where M is a transition metal, A is an A group (IIIA or IVA) element, and X is C or N, and n varies from 1 to 3 (Ghosh and Harimkar, 2012). They are polycrystalline with ternary carbides and nitrides. MAX alloying and 2D MAX phases, *MXenes*, have been extensively explored for different kinds of application, including but not limited to battery storage applications (Naguib et al., 2013; Zhao et al., 2019a), catalyst, coating for corrosion resistance or thermal stability, cladding, and magnetic application (Li et al., 2020; Lu et al., 2017; Novoselova et al., 2018). Selection of synthesizing route and process change can determine the morphology of MAX phases properties and application (Wang et al., 2017). Mechanically milled Cu with Ti_3_AlC_2_- vol. 40% Cu powder mixture up to 10h followed with hot pressing at 950°C and 25 MPa for 1h. Alloying of Cu is mainly observed at Al (A Site). The resultant structure of Ti_3_AlC_2_ – Cu(Al) composite exhibits a low resistance and high compressive strength ∼1240 MPa because of decreased Ti_3_AlC_2_ crystallite size and microstructure homogeneity improve mechanism (Nechiche et al., 2017). Furthermore, mechanically milled Ti_3_AlC_2_-40 vol.% Cu powder mixture for 20 min, and cold compacted at 300 MPa, followed by sintering at 760°C for 2h. As a result, Cu was alloyed with Al with ∼50% composition and Ti with ∼3%. Selective etching was performed to dissolve composite Cu (Al, Ti) to get the solid solution. It showed (Ti_(1-x)_ Cu_x_)(Al, Cu) that C_2_ resulted in a higher lattice parameter, which may indicate distortion in the lattice. Ti_3_SiC_2_ MAX Phase is intrinsically self-lubricating properties and TiC second phase for imparting strength (Magnus et al., 2020), which means that the Ti_3_SiC_2_ can be easily dry ball milled for further alloying. Most of the attempts are synthesizing through powder metallurgy, which mainly depends upon either material self-lubricating properties or the addition of lubricating material for mechanical milling to avoid any foreign inclusion due to wear and tear (Haji Amiri et al., 2020; Ma et al., 2020; Wang et al., 2020b). There are also novel approaches recommended for formation, such as the arc-melting method, PVD and laser treatment, and molten salt route (Bahiraei et al., 2020; Fattahi and Zarezadeh Mehrizi, 2020; Galvin et al., 2018; Tunca et al., 2020). Wang et al. explored applying the Cr_2_AlC MAX phase as a coating using the arc/sputtering deposition method (Wang et al., 2020c). The coating showed good oxidation resistance at temperatures as high as 1100°C due to the Al_2_O_3_ interface layer and (Cr, Al)_2_O_3_ at the outermost layer (Figure 5C). Similar phenomena were reported for 211 and 413 V-Al-C MAX phase corrosion resistance (Ghasali et al., 2021, p. 211). Application of coating can also impart additional mechanical properties. The Ti-Al-C MAX phase can be rapidly formed from elemental powder, and the presence of the Ti_2_AlC, TiC_x_, and Ti_x_Al may provide surface hardness of 811 Vickers hardness (Richardson et al., 2020). Another application of MAX phase coating (Akter et al., 2020) recommends that Hf_2_SN and Hf_2_Sc are good candidates to decrease solar heating. A recent study shows the problem of *MXenes*, the rate at which *MXenes* oxidize and degrade as stored as an aqueous suspension (Mathis et al., 2021). The experiment suggested that adding excess aluminum, Al, during synthesis leads to improved crystallinity of M*Xene* that can exhibit an increased shelf life ten times. This study supports the usefulness of an extensive study done on *MXenes*. (Halim et al., 2018)showed etching using HF leads to M*Xene* formation from MAX phase because of weak interlayer connection between M and A atoms (Figure 5D). Study that synthesized (Nb_2/3_ Sc_1/3_)_2_AlC quaternary system and selective etching removed Sc and Al atoms, leading to the formation of Nb_1/3_C. Mo_2_ScAlC_2_ suggested the existence of new quaternary MAX phase alloys with out-of-plane chemical order; Mo_2_ScAlC_2_ with an Sc atoms layer sandwiched between two Mo-C layers. MAX phase selective etching of Al using HF leads to formation of M*Xene* Mo_2_ScC_2_ (Meshkian et al., 2017). (Aghamohammadi et al., 2018) studied the Ti_3_SiC_2_ MAX phase immersion in Hydrofluoric acid (HF). The result showed that the Si layer disappeared and broke down to TiC. In addition, it changed the morphology of TiC, before TiC had low crystallinity, but after HF Treatment, TiC was shaped to hexagonal and then to truncated octahedron and cubic. (Persson et al., 2018)showed the formation of *MXenes* from the *i-*MAX phases. (Mo_2/3_Y_1/3_)_2_AlC *i*-MAX phase was selective etched using HF, which led to the removal of Al atoms and formation of (Mo_2/3_Y_1/3_)_2_C and M_1/3_C *MXenes. MXenes* application was further explored in the study. (Mo_2/3_Y_1/3_)_2_C showed better supercapacitor performance when used as an electrode with KOH electrolyte, whereas M_1/3_C showed higher capacitance with H_2_SO_4_ solution (Chirica et al., 2021; Zhao et al., 2021). Selective etching of hybrid A layers (Al, Cu) resulted into Ti_3_(Al_(1-x)_ Cu_x_)C_2_ M*Xene*, where its application was explored as a catalyst to efficiently electro-reduce CO_2_ into methanol. Another application of selective etching in the MAX phase was attempted to find its application in batteries. Ti_x_Ta_(4-x)_AlC_3_ was selective etched using HF and resulted into Ti_x_Ta_(4-x)_C_3_ MXene. Transformation promoted its structural delamination with an expanded interlayer d-spacing, allowing effective reversible Li-ion storage (Syamsai et al., 2021). (Näslund et al., 2020) show delocalized electron redistribution from Ti_3_C_2_ to Al layers in Ti_3_AlC_2_ electrostatic interaction. Selective etching may lead to M*Xene* compound formation. The previous study shows 211 phase formation in (Zr,Nb)_2_(Al,Sn)C MAX phase quaternary system. They hypothesize that comparable atomic radii of M and A atoms obtain a stable 211 MAX phase (Lapauw et al., 2018). As a result, adding Nb, Sn reduces distortion of lattice parameter compared to pure ZrAlC and found two quaternary systems, (Zr Nb)_2_AlC and Zr_2_(Al Sn)C, stable over their entire compositional range. Similar phenomena were observed for (Zr Ti)_2_ (Al,Sn)C MAX phase quaternary system, where Sn atoms alloying with A atoms help to achieve phase purity, and Ti atoms alloying with M atoms lead to a decrease in lattice parameters. (Tunca et al., 2019) explored the semi-conducting application of h-BN *via* fluorinating using Nafion, a perfluorinated polymer (Figure 5E) (Radhakrishnan et al., 2017). The fluorination process showed fluorine (F) radicals interacting with B-N bonds and significantly change the charge density of nitrogen atoms, resulting in ferromagnetic ordering and widening its bandgap. This simple approach can be fruitfully used for the fluorination of other 2D materials.

## Limitations of the study

### Utilization of phase diagrams

Phase diagram plays a very crucial role in the development of materials. Utilizing the phase diagram, we can know the temperature, phase boundaries, and solubility of the target phase. In addition, it can help decide phase composition in the alloy system as we tend to explore multicomponent (binary, ternary, and more); it is crucial to know the phase diagram of the system. Unfortunately, there is limited information (phase diagram) available for most of TMDCs. Hence, building a thermodynamic database, possibly via combining first principle with mesoscale calculation for a set of possible molecular combinations, to construct a phase diagram will be crucial for future TMDCs development. Such computational-assisted phase diagram database would offer monumental potential for the discovery and innovation of new materials.

### Development of energy-efficient methods to alloy elements in TMDCs

In literature, the isomorphs substitution in alloy TMDCs explores in greater detail. They are relatively easy to synthesize owing to extended solubility in each other. On the other hand, the non-isomorphous elements are difficult to alloy, as they need extreme conditions. Taking inspiration from nature, the alloying happens with minimum energy input. In the future, new synthesis routes to alloy elements in energy-efficient methods will be crucial. It can be bio-assisted synthesis methods or energetically stabilized alloys systems, or anything else.

### Improving mechanical properties and environmental stability

The recent reports show limited stability of the 2D TMDCs, which can be further improved with the help of alloying elements. In the materials development program of structural alloys (e.g., Ni-based superalloys), appropriate alloying elements are added to improve its oxidation/corrosion resistance. The idea can be extended to improve the environmental stability of the TMDCs. Alloying improves/tunes the mechanical properties of materials, in general. The similar concept can be extended in 2D materials as well. There are recent work utilizing such aspect, but most of the mechanical testing is indirect. The *in-situ* mechanical testing either with Raman or electron microscope will be helpful. Quantifying the mechanical properties will be crucial for future automation in device fabrication or large-scale transfer.

### Utilization of machine learning

Alloying in 2D materials provides enormous opportunity to design new 2D materials consisting of new structure or phases. It also changes the physical and chemical properties drastically. In such case, selection of required 2D materials with specific structure/phase with certain required physical and chemical properties becomes a difficult task. Synthesizing multiple 2D materials and measuring the properties are challenging. In such case, machine learning (ML) can be utilized. Employing machine learning tools to materials design or optimizing materials processes, etc., critically depend on the accuracy of the machine learning parameters used. Inaccuracy or unreliable parameter values would invariably ingress errors in the prediction and lead to unreliable prediction. Thus, care must be given in reliably selecting the parameters carefully. Physics-based machine learning approach as compared to curve fitting or empirical approach would yield higher reliability of using machine learning methodologies in materials discovery and design. There are few efforts to utilized ML for synthesis of single component 2D or single phase TMDs. In future, it can be utilized for design of alloy 2D materials with specific composition and physical/chemical properties.
